# Vaccination-induced changes in human B-cell repertoire and pneumococcal IgM and IgA antibody at different ages

**DOI:** 10.1111/j.1474-9726.2011.00732.x

**Published:** 2011-12

**Authors:** Alexander Ademokun, Yu-Chang Wu, Victoria Martin, Rajive Mitra, Ulrich Sack, Helen Baxendale, David Kipling, Deborah K Dunn-Walters

**Affiliations:** 1 Peter Gorer Department of Immunobiology, King's College London School of MedicineLondon SE1 9RT, UK; 2 Lambeth Walk Group PracticeLondon SE11 6SP, UK; 3 Institute of Clinical Immunology Medical Faculty, University of LeipzigLeipzig, Germany; 4 Papworth Hospital NHS Foundation TrustCambridge CB23 3RE, UK; 5 School of Medicine, Cardiff UniversityCardiff CF14 4XN, UK

**Keywords:** ageing, B-cell repertoire, immunoglobulin A, immunoglobulin M, vaccination

## Abstract

It is well known that older people are more susceptible to morbidity and mortality from infectious diseases, particularly from pulmonary diseases such as pneumococcal pneumonia where vaccines do not provide efficient protection as in younger populations. We have previously shown that the B-cell repertoire in the old is reduced and hypothesise that this may contribute to the impaired humoral responses of the elderly. Here, we investigated the repertoire and antibody responses to winter vaccination in two age groups, aged 18–49 and 65–89. We found that the serum IgM and IgA pneumococcal responses were significantly impaired in the older group, with no difference in IgG levels. *IGHM* spectratype analysis seems to be the most promising in terms of its predictive ability for vaccine responses. Spectratypes showed a clear change in the repertoire at day 7 after vaccination, with a return to the baseline levels at day 28. The changes at day 7 reflected expansion of *IGH* sequences that have smaller, more hydrophilic, CDR3 regions, and these changes were attenuated in the older group. The older group was more likely to have spectratypes indicative of a reduced diversity at day 0 and day 28. On average, the baseline repertoire in the older group was comprised of larger CDR3 regions than in the younger group. In conclusion, IgA and IgM responses are significantly impaired in the elderly pneumococcal response and are likely key mediators of protection. Hydrophilicity and/or small size of the *IGH* CDR3 appear to be important in these responses.

## Introduction

*Streptococcus pneumoniae* causes significant morbidity and mortality in young children and in older adults, and it is the most significant secondary infection associated with influenza. Many deaths attributed to influenza may actually be caused by secondary pneumococcal infection (van der Sluijs *et al.*, 2004). Vaccination against pneumonia has proven difficult in these high-risk groups, where the standard 23-valent pneumococcal polysaccharide (PPS) vaccine has not been as effective as in healthy young adults (Moberley *et al.*, 2008). Conjugate protein–PPS vaccines have had more success in the very young but do not seem to improve vaccination responses in the old (Baxendale *et al.*, 2010). The overall picture of antibody responses in old age seems to be one of changes in specificity and quality rather than a decrease in quantity (Howard *et al.*, 2006). While the efficacy of vaccination against both influenza and pneumonia decreases significantly with age, the levels of poly-specific and autoreactive antibodies increase with age. It has been reported that vaccine challenge in the older population can actually increase levels of anti-DNA autoantibodies, implying an age-related loss of focus in the immune response (Huang *et al.*, 1992). A lack of focus in affinity maturation of antibodies has been implied by the fact that the B cells in germinal centre responses in Peyer's patches of older people appear to be under less selection pressure than in younger samples, although this may be a tissue-specific phenomenon (Banerjee *et al.*, 2002).

Specific IgG antibody levels are sometimes used as a correlate of protection in assessing vaccine responses, although this may not always be the best measure. It has been shown for PPS IgG responses that the actual levels of PPS-specific IgG do not always correlate with the functional opsonophagocytic assay (Anttila *et al.*, 1999). While IgG is by far the most abundant antibody produced in vaccine responses in general, we do not believe it is always the most relevant. When considering the most common site of infection by pneumococcus, natural immunity against colonisation and pneumonia is most likely provided by antibodies of IgA and IgM isotype which are generated within the mucosal-associated lymphoid tissue. In the case of the PPS vaccine, the antigen is polysaccharide, a type II T-cell-independent (TI2) antigen. TI2 responses differ from T-dependent (TD) responses in many respects, and it has been suggested that these two responses might be differentially affected with ageing (Frasca *et al.*, 2007). IgM memory B cells have been shown to be important in providing early defence against pneumococcal infection, and the loss of IgM-producing B cells has been shown to impair the potential to generate adaptive immune responses (Boes *et al.*, 1998; Kruetzmann *et al.*, 2003). Postvaccine IgM antibodies with characteristics of natural antibodies can be protective against invasive pneumococcal disease (Baxendale *et al.*, 2008). Although some reports do not find a change in levels of IgM memory cells with age (Colonna-Romano *et al.*, 2006), others have shown an age-related decrease in IgM memory cells with age and indicated that induction of IgM responses in the elderly might be impaired (Shi *et al.*, 2005). The best evidence for a key role of IgM in pneumococcal protection is a recent report that depletion of IgM antibodies from the serum severely reduces opsonophagocytic activity against two common PPS serotypes (Park & Nahm, 2011). IgM is extremely efficient at opsonisation compared with IgG; therefore, even a small age-related change in IgM levels may be significant for efficacy of vaccines in old age.

Antibody genes can be used to measure the diversity of different B-cell populations, and there have been some reports that gene usage against specific PPS serotypes may vary with age (Kolibab *et al.*, 2005). These data are not directly relevant to a study of IgM and IgA PPS responses, because they are limited to only two of the six *IGHV* gene families of the IgG isotype against two PPS serotypes. However, they do indicate that an immune failure to respond to PPS antigens may have origins in an altered B-cell repertoire. Immunoglobulin heavy chain sequences are comprised of three different genes (*IGHV, IGHD and IGHJ*) in conjunction with a constant region, of either alpha, gamma, mu or epsilon, that determines the class of antibody produced. The *IGHV–IGHD–IGHJ* junctional region of the gene is known as the CDR3 region and is highly diverse. It is this region that is thought to be most important in the antigen-binding pocket of the antibody (Kirkham & Schroeder, 1994). The diversity is such that in a normal population of B cells, there will be a Gaussian distribution of CDR3 sizes, which can be visualised by separation of fragments electrophoretically to produce a spectratype. Departure from a Gaussian distribution is taken to indicate perturbation of a repertoire by clonal expansion of cells of a particular CDR3 size. Although B-cell spectratypes have previously been used to measure perturbations in diversity of repertoire through old age (Gibson *et al.*, 2009), or through disease (Foreman *et al.*, 2008), there has been no systematic spectratype study to determine how the repertoire of different peripheral blood B cells changes after vaccination.

We have previously shown that a DNA spectratype can show a reduced diversity of B-cell repertoire in older people and that this is correlated with frailty (Gibson *et al.*, 2009). It is thought that a reduced diversity is a consequence of a decreased naïve B-cell input from the bone marrow and therefore, within the homeostatic B-cell environment, a lack of contraction of antigen-experienced B-cell expansions to maintain the overall B-cell number in the periphery (Cancro *et al.*, 2009). We hypothesised that a reduced B-cell diversity in the first instance might be the cause of a reduced immune response to challenge in older people, there being less novel naïve cells to respond to challenge. To test this hypothesis, we investigated the effect of vaccination on PPS-specific serum IgM, IgA, as well as IgG antibodies, and on cDNA-derived spectratypes for the corresponding *IGHM*, *IGHA* and *IGHG* classes.

## Results

### Spectratype analysis shows challenge-related changes

**Fig. 1:**
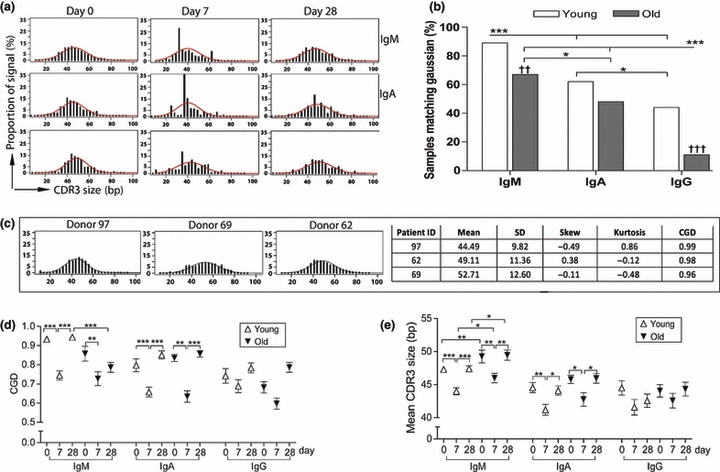
B-cell CDR3 spectratypes show individual variability and show challenge-related changes. (a) Spectratypes of samples from one individual before, and at 7 and 28 days after, receiving winter vaccination for influenza and pneumonia, showing CDR3 size distribution histograms for the *IGHM, IGHA* and *IGHG* immunoglobulin genes in peripheral blood B cells. The best fit Gaussian distribution curve for each sample, as determined based on the mean and SD values, is overlaid. The *X*-axis represents the CDR3 size in nucleotides, and the *Y*-axis is the proportion of the CDR3 sequences at each size. (b) Spectratype data show that diversity of repertoire decreases with age and with change from IGHM to switched isotypes on day 0 before challenge. High correlation to the underlying Gaussian distribution (CGD) is an indicator of a high level of diversity in the population. The *Y*-axis shows the per cent of spectratypes that have a correlation value of over 0.9 in each group. Variables were compared using chi-squared test with Bonferroni post-test, and significant values for comparisons between isotypes are indicated using * and between ages are indicated by †. (c) CDR3 spectratypes can follow a Gaussian distribution but may still differ between individuals. Examples of *IGHM* spectratypes from three different young individuals before vaccination are shown, with differing mean, SD, skew, kurtosis and CGD values. (d) Challenge and age-related changes in repertoire diversity as indicated by the spectratype correlation to the baseline CGD in the young and old age groups of different isotypes before, and at 7 and 28 days after vaccination. Groups were compared using Mann–Whitney *u*-test. (e) Challenge and age-related changes of the mean CDR3 length. Groups were compared using Mann–Whitney *u*-test. Error bars are SEM, *P < 0.01, **P < 0.001 and ***P < 0.0001.

To determine whether we could detect vaccination-related changes in B-cell repertoire, we used primers specific for *IGHM*, *IGHA* and *IGHG* classes, together with an *IGHV* framework 3-specific primer, to amplify the CDR3 region of Ig genes in an isotype-specific manner. Samples were of cDNA from peripheral blood lymphocytes at day 0, immediately before vaccination, and at days 7 and 28 after vaccination. The resulting fragments were separated by high-resolution electrophoresis to produce spectratypes, and after normalising all the samples, the mean and standard deviation (SD) values were used to determine the underlying Gaussian distribution for each spectratype (Fig. 1a).

At day 0, the *IGHM* spectratypes were generally a better fit to a Gaussian distribution than *IGHA* spectratypes and the samples in the young group were a better fit than in the old group (Fig. 1b). As expected, there is interindividual variability in spectratypes, and this is especially pronounced in *IGHA* and *IGHG*, presumably due to the fact that switched samples reflect prior antigen experience and will therefore contain expanded populations of cells that reflect the individual's prior exposure. However, even in the day 0 *IGHM* samples that closely approximate Gaussian distributions, there are interindividual differences in repertoire evidenced by differences in mean, SD, skewness and kurtosis (Fig. 1c). As the *IGHM* spectratype reflects a population of cells where the majority (approximately 85%) are naïve cells, then this implies that the baseline naïve B-cell repertoire varies on an individual basis.

In both young and old samples, there is a striking change in the repertoire at day 7 after challenge with vaccine. The spectratypes change at day 7 in all three isotypes and return to shapes more similar to the day 0 baseline for that individual by day 28. This is seen more easily in the smoother spectratypes of *IGHM* than in those of the more variable *IGHA* and *IGHG* isotypes (Fig. 1a). In particular, the pre-existing clonal expansions of IgG meant that the background noise in the data prevented the detection of vaccine or age-induced changes of IgG spectratypes at this sample size. As a perturbed B-cell repertoire can be changed in several respects, with increases or decreases in peaks at different points across the spectratype for each individual, then the overall postchallenge changes in spectratypes are best illustrated by measurements of the correlation of the sample spectratype to the baseline Gaussian distribution (CGD), as seen in Fig. 1d. The CGD decreases at day 7 and returns to the higher value at day 28. Although the marked variability in *IGHG* values masks any differences that might exist between day 0 and 7 in either age group, there was a significant change between day 0 and 7 in the *IGHM* and *IGHA* spectratypes in both age groups.

Evidence in support of the notion that changes in response to antigen challenge are influenced by particular characteristics of the Ig genes involved is shown by highly significant changes in the mean values of the spectratypes, which reveal evidence for strong selective pressure in favour of a smaller CDR3 region in the responding population (Fig. 1e). Both groups showed a significant decrease in mean CDR3 length between day 0 and 7 in *IGHM* and *IGHA* spectratypes. Most notable is the fact that the old group has longer CDR3 regions than the young group, with significantly higher mean CDR3 values for *IGHM* genes at all three time points, indicating that the older people have longer CDR3 lengths even at baseline in the absence of challenge.

### Sequence analysis indicates a change in the underlying repertoire as well as expanded B-cell populations

**Fig. 2:**
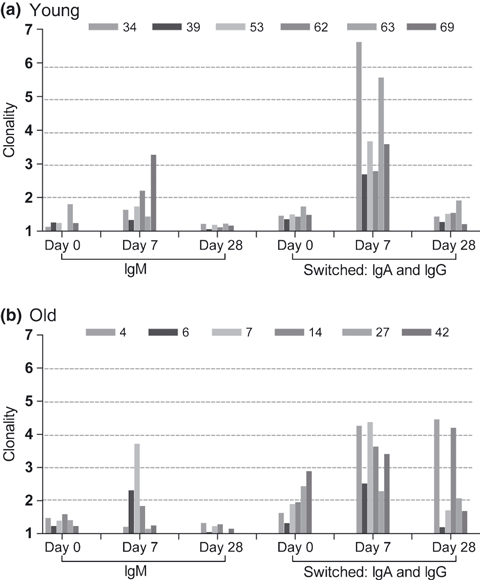
Challenge-induced B-cell clonality changes. Clonality was determined by dividing the overall number of sequences by the number of different clonotypes (defined by CDR3 region identity). Data represent day 0, 7 and 28 for (a) six young samples (b) and six old samples. Data are plotted separately to indicate interindividual variation, as indicated by different bar shading.

To confirm the hypothesis that the spiky characteristics (low CGD values) of the day 7 cDNA-based spectratypes reflect a clonal expansion of antigen-specific B cells, we used high-throughput sequencing to obtain 36 935 immunoglobulin sequences from PBMC samples at days 0, 7 and 28 from six old (aged 70–89) and six young (aged 19–45) individuals. Sequences that belonged to the same clone family were identified by shared CDR3 regions (Table 1). Figure 2 clearly shows that although there is considerable interindividual variability, clonal expansions occur at day 7 and to a greater extent in switched isotypes than in *IGHM*. There is still some evidence of clonality at day 0 and 28, even in the *IGHM* populations. The older group in particular contained some individuals with considerable clonality of switched sequences at days 0 and 28, indicating a lack of diversity even in the absence of immediate antigen challenge.

**Table 1:** The numbers of immunoglobulin CDR3 sequences from each individual

			No. of all sequences		No. of unique clonotypes*	
				
Groups	Donor ID	Age (years)	IgA and IgG	IgM	IgA and IgG	IgM
Young	34	22	4704	978	1505	758
	39	19	1454	608	953	490
	53	22	2335	1011	1075	752
	62	33	946	39	471	31
	63	22	5157	689	1853	448
	69	45	1026	388	398	175
Old	4	71	5201	558	1588	426
	6	76	901	845	442	567
	7	87	1610	92	727	57
	14	70	3823	550	1052	329
	27	89	1035	53	454	42
	42	73	2519	413	885	343
	Total	–	30711	6224	11403	4418

* Sequences were grouped into clone families by similarity of DNA sequence, and one example clonotype from each family was counted. As different primers were used to isolate the different isotypes, the relative numbers of IgM/IgG/IgA sequences cannot be taken to reflect the relative numbers occurring *in vivo*.

**Fig. 3:**
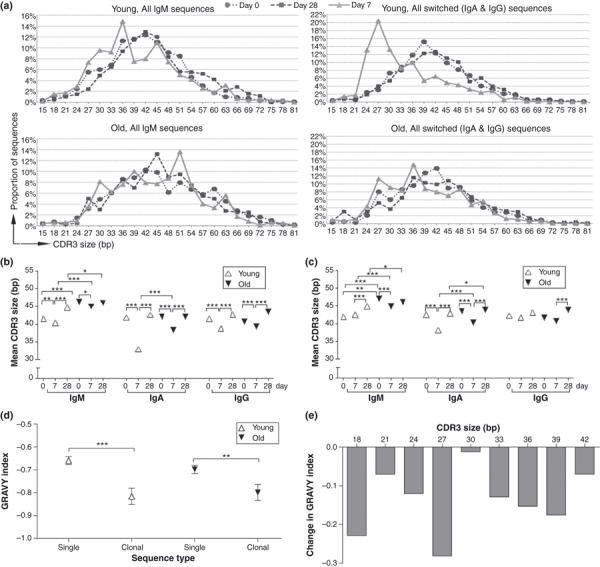
Challenge results in selection of smaller, more hydrophilic, CDR3 regions. (a) Virtual spectratypes were created from sequencing data by plotting the distribution of different sized CDR3 regions for all the IGH sequences obtained. Curves represent day 0 (circles), day 7 (triangles) and day 28 (squares). The underlying distribution, without the clonal expansion effect, can be seen by plotting the distribution of CDR3 size when only one sequence from each clonotype is counted (Fig. S1). The mean CDR3 sizes for all sequences are shown in (b), while the mean CDR3 sizes for unique clonotypes are shown in (c). (d) For smaller sequences, where the CDR3 size was 42bp or less, the Grand Average Hydrophobicity values (GRAVY index) were plotted for sequences that were found only once (single) and that were found in related clones where there were more than three sequences per clone (clonal). Only one example sequence from each clone was included to avoid bias because of clonal expansion. (e) Illustrates the difference in average GRAVY values for single vs. clonal sequences at each different CDR3 size. At all sizes, the values for clonal sequences are lower and more hydrophilic, than those for single sequences. Comparisons were performed by Mann–Whitney *u*-test. Error bars are SEM, *P < 0.01, **P < 0.001 and ***P < 0.0001.

Sequence data can be displayed to produce ‘virtual spectratypes’. These have the advantage of being able to visualise the distinction between an underlying repertoire perturbation and a change because of clonal expansion of B cells. The CDR3 sizes of all sequences were counted, and the frequency distribution was plotted to give a virtual spectratype of all sequences (Fig. 3a). In agreement with the data from the polymerase chain reaction (PCR) spectratypes described earlier, in the young group, there is a substantial shift towards a smaller CDR3 size at day 7 after challenge, while days 0 and 28 are generally similar to each other (Fig. 3a,b). The day 28 values for IgM appear to reach a higher CDR3 length than day 0, which are not in agreement with the data from the spectratypes in Fig. 1. As the sequence data are obtained from only six subjects per group, these are more affected by interindividual variation than the spectratype data. It is possible that one of the six subjects had an influx of IgM-containing B cells with longer CDR3s (such as transitional B cells, as reported by Wu *et al.*, 2010) that could have caused this discrepancy. The old group has a more subtle shift at day 7, and there is a significant difference in CDR3 size between young and old in *IGHM* at all time points, and in *IGHA* at day 7 (Fig. 3b). To explore whether clonal expansion was a dominant effect accounting for the age- and time-related differences, these virtual spectratypes were plotted using unique Ig gene sequences (clonotypes), to remove the effect of clonal expansion. Young and old had an equivalent response in terms of the change in CDR3 size at day 7 postimmunisation (Fig. S1). However, the age-related differences between overall CDR3 size values remained significant (Fig. 3c).

We investigated whether clonal expansions in the different age groups occurred in particular sequences by determination of clone sizes at the different CDR3 sizes for both young and old groups. Larger clone sizes correlate with lower CDR3 sizes in the young group only (Fig. S2). Analysis of the unique sequences from the younger group revealed a significant positive correlation between CDR3 size and hydrophobicity (r^2^ = 0.017, *P* < 0.0001, Fig. S3). We also saw a significant decrease in hydrophobicity in expanded clones when compared with single sequences in the below average CDR3 size range (Fig. 3d). This selection for decreased hydrophobicity in responding clones was not because of any one particular CDR3 size (Fig. 3e).

### Serology

**Fig. 4:**
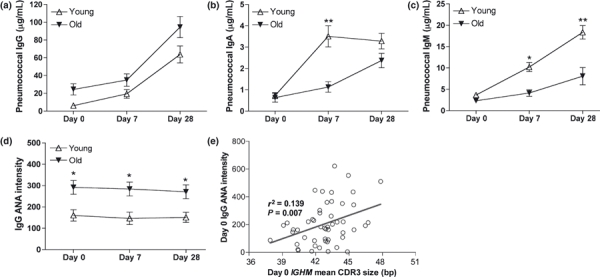
Antibody responses to vaccination. ELISA was performed using a pool of seven pneumococcal polysaccharides (4, 6B, 9, 14, 18C, 19F and 23F) and specific for (a) IgG, (b) IgA or (c) IgM antibodies. The levels of IgG antinuclear antibody were also determined at each time point (d), and we found a significant correlation between the mean CDR3 size of the *IGHM* spectratype at day 0 and the levels of IGANA at day 0 (e). Comparisons were performed by Mann–Whitney *u*-test. Correlation by Pearson's test. Error bars are SEM, *P < 0.01, **P < 0.001 and ***P < 0.0001.

The serum response to immunisation was characterised by pneumococcal PS-specific ELISA. To facilitate comparison of C-PS antibody responses with multiple metrics, we used a pooled ELISA incorporating seven key serotypes (4, 6B, 9, 14, 18C, 19F and 23F). IgG was detected at higher concentration than IgA or IgM, and the older cohort had higher concentrations of IgG pre-immunisation relative to younger adults, although this difference was not significant (Fig. 4a). A rise in serum IgG, IgA and IgM was seen in all individuals postimmunisation; however, the time course was different by isotype and varied between the two cohorts. IgG serum responses were similar between young and old, peaking at day 28, consistent with data from other groups (Lee *et al.*, 2010; Park & Nahm, 2011). In contrast, in the young individuals, an early peak IgA serum response was detected at day 7, while in the older cohort, the rise in IgA was more gradual, peaking at day 28 (Fig. 4b). The serum IgM response to immunisation was reduced in the older cohort at days 7 and 28 postimmunisation (Fig. 4c).

As it has been reported that levels of autoantibodies can also increase as a result of vaccine challenge (Huang *et al.*, 1992), we measured the levels of IgG antinuclear antibodies (IgG ANA) before and after vaccination using a HEp2 slide-based cytometric assay (Hiemann *et al.*, 2007, 2009). It can be seen from Fig. 4d that as expected, there is an age-related difference in the levels of IgG ANA; however, this is unchanged by vaccine challenge. Curiously, we saw a significant correlation between longer CDR3 size and autoantibody IgG ANA at day 0 (Fig. 4e). This may be an indirect relationship, because both values correlate with age, although there was still a significant correlation when only the over 65s are considered (*r*^2^ = 0.2045, *P* = 0.0303).

### Ig repertoire correlates with antibody response

Given that there were significant age- and challenge-related changes in the metrics associated with the B-cell spectratypes, we hypothesised that there would be a relationship between the serum antibody response and spectratype data.

Leptokurtosis (higher peakedness) was significantly correlated with higher PPS-specific serum IgA values but did not quite reach significance when compared with IgM values (Fig. S4a,c). Small SD values of spectratypes at day 7 were correlated with both higher PPS-specific serum IgM and IgA values (Fig. S4b,d). As the predictive value of spectratypes would be of much more value if we could predict the vaccine response prior to administering the vaccine, we looked for correlations between the peak PPS-specific IgA and IgM responses and the spectratype metrics at day 0. Despite the fact that the mean CDR3 size values of the *IGHM* spectratypes had shown significant age-related differences, correlations between the *IGHM* day 0 values and serum IgA, IgG and IgM levels did not reach significance (Fig. S5).

**Fig. 5:**
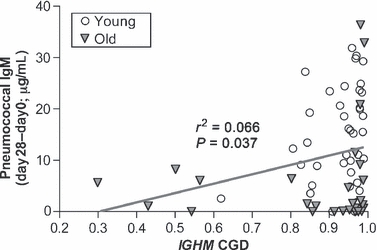
*IGHM* spectratype before vaccination has some predictive ability. The increase in IgM pneumococcal polysaccharide-specific antibody at day 28 shows a significant correlation with the CGD index of the *IGHM* spectratype. Samples in the young group are plotted as open circles, those in the old group as solid triangles. Correlation by Pearson's test.

We hypothesised that a less diverse repertoire would predict a worse immune response and therefore looked to see whether CGD at day 0 can predict the peak antibody response. There did not appear to be any simple correlative relationship between spectratype metrics and serology for the IgA and IgG responses, although there were indications that the spectratypes and antibody responses were related. In *IGHM* in particular, the best PPS-specific IgM antibody responses at day 28 were indeed associated with a CGD value nearer to 1 (i.e. a more diverse repertoire), with a value below 0.8 predictive of a poor antibody response (Fig. 5, *r*^2^ = 0.06574, *P* = 0.037). However, having a CGD value > 0.8 does not guarantee a good IgM antibody response at any age. It could be seen that the younger group was more tolerant of a loss of diversity. A day 0 *IGHM* spectratype CGD value of lower than 0.9, together with age over 65, indicated a worse IgM response.

## Discussion

We observed that even in the absence of challenge, there are differences in the B-cell spectratypes between individuals (Fig. 1). These can represent changes in B-cell repertoire because of prior antigen exposure or changes in the naïve repertoire. With regard to the latter, the *IGHM* spectratypes are particularly interesting, because the assumption is that they largely represent naïve B cells. This would imply that there is an element of interindividual variation in factors affecting repertoire generation in central B-cell development. These could be factors related to gene rearrangement, such as preferential use of different *IGH* genes or terminal deoxynucleotidyl activity. Alternatively, they could be factors associated with tolerance selection of the repertoire such as occurs in the bone marrow and in the transitional B-cell stages (Wardemann *et al.*, 2003). These factors may also be responsible for the age-related differences in *IGHM* CDR3 size that we see before vaccine challenge.

Vaccine challenge results in cellular changes in the peripheral blood at day 7 (Wrammert *et al.*, 2008; Baxendale *et al.*, 2010). These can be detected both by spectratype metrics showing a decreased diversity (lower CGD value of spectratype) and by sequence analysis indicating an increase in clonality. Although the sequence analysis only compared six old and six young, and there was interindividual variability (Fig. 2), we could still show a decrease in the extent of clonal expansion at day 7 in the old compared with the young. The older group also has less diverse repertoires at days 0 and 28, and they do not return to day 0 CGD values at day 28 as the younger group do. This may be related to the slower time to reach the peak IgA antibody response in the serum. Without the benefit of further time points, it is difficult to judge the kinetics of the response. It is entirely possible from these data that the IgA response in the old follows the same pattern as the young but that it is delayed as a whole so that it peaks later and resolves even later. Alternatively, there may be different factors affected in the activation and resolution phases of the response to cause these observations. Inadequate resolution of immune responses may be just as important as a failure to respond in the first place, and although literature is sparse on this topic, there are indications that regulatory functions of cells may be impaired with age (Hwang *et al.*, 2009). A delayed resolution could be a significant contributory factor to the observations that older people have less diverse repertoires, because if the effects of a single challenge can still be seen a month later, then the multiple challenges we encounter as part of everyday life could present a significant compromise to the baseline diversity.

While we have shown that we can use spectratyping of the B-cell repertoire in an isotype-specific manner, it is clear that the high levels of interindividual variability in prior antigen exposure are such that it would not be useful as a predictor in a study of IgG responses. The subjects in the study had both influenza and pneumococcal vaccinations, as part of their normal healthcare, thus were exposed to multiple antigens simultaneously. In spite of the complicated challenge, there are indications that some measures of the spectratypes can be correlated with the PPS-specific responses. The serum IgM and IgA antibody levels at day 28 were associated with more leptokurtotic *IGHM* and *IGHA* spectratypes at day 7. This peakedness seems to be a result of the expansion of clones containing smaller CDR3 values. In looking for a prevaccine predictor, we could see promising associations between the spectratype shape (CGD, Fig. 5) and the IgM response. There was a significant difference in CGD values between the young and the old groups, and the individuals with the highest levels of IgM have CGD values near to 1. If an individual is in the over 65 age bracket and has a CGD value of lower than 0.9, then they will not produce a high level of PPS-specific IgM antibody.

It is clear, from data here and elsewhere, that the IgM response is severely diminished in the older population. Whether this is because of a failure of the TD or the TI pathway of B-cell activation cannot be determined from these data, but the nature of the PPV23 vaccine would lead one to expect the latter. In the light of these data, and the recent report that removal of IgM antibody from serum diminishes opsonophagocytic activity against two common PPS serotypes (Park & Nahm, 2011), it would seem appropriate that future studies aimed at improving PPS responses in the elderly should include consideration of the IgM response. In addition, we found that although the IgA serum response was not significantly different in the two age groups at day 28, there was a significant difference at day 7 (Fig. 4). This implies that there might be an age-related temporal difference in either production or trafficking of IgA. Temporal differences such as this in the face of bacterial infection could have severe consequences for an individual, and these data have highlighted the need for analysis at different time points after vaccination. Although we saw a significant increase in IgG ANA autoantibodies with age, we did not see any evidence that vaccination caused an increase as has previously been reported (Huang *et al.*, 1992). This may be due to differences in adjuvant use in vaccines, but it is comforting to know that the recent commonly used vaccines have no deleterious effects in this respect.

Perhaps the most striking observation from these spectratype data is that the mean size of the CDR3 region is significantly decreased after challenge in both young and old age groups for all isotypes. The extent of the change in the CDR3 size appears to be the same for both age groups, but the old group starts at a higher size in the first instance, so that there are significant age-related differences in CDR3 size both before and after vaccination (Fig. 1e). The precise effect of these differences is not known, because we could not find any clear correlations between CDR3 size and serum antibody. The lack of correlations with antibody might reflect the noise in the experiment caused by having multiple vaccine challenges at the same time, or it may be due to some other factor such as differential locations for responding cells vs. responding antibody. The sequencing data confirm the spectratype data, showing an overall decrease in mean CDR3 size, and also show that when the effect of clonal expansion is removed the challenge-induced CDR3 size shift is similar between the two groups. Selection for smaller CDR3 size has previously been reported in the germinal centre response to hapten NP in mice (Lacroix-Desmazes *et al.*, 1998), and a study of 256 peripheral blood *IGHV6-1* IgM genes in humans has noted that antigen-experienced genes have shorter CDR3 than those that are unmutated (Rosner *et al.*, 2001). More recently, we have shown for the whole repertoire of *IGHV* genes in three young individuals that memory B cells have shorter CDR3 regions than naïve or transitional B cells (Wu *et al.*, 2010). This is also apparent in these data, where class-switched (and therefore antigen-experienced) genes have shorter CDR3 regions than IgM genes even at day 0. A small CDR3 size appears to be important in relation to the hydrophobic nature of the antigen-binding site of the antibody; lower sizes of CDR3 regions tend to have a lower hydrophobicity and within this, even when sequences of the same size are compared, those that belong to expanded clones have a further decrease in hydrophobicity compared with the unique sequences. So it looks very much as if sequences with a smaller, more hydrophilic, antigen-binding site are being selected and expanded in the younger population more so than in the old.

In summary, even though the level of serum IgA and IgM antibodies is much lower than that of IgG antibodies, it is in the two former isotypes that the significant age-related defects in the response to pneumococcal vaccine are found. B-cell spectratype analysis of *IGHM* sequences seems to be the most promising avenue for predicting responses to pneumococcal vaccination, although it appears that there are still factors other than B-cell repertoire that are involved in the senescence of the humoral immune response. We have also shown clear evidence that expansion of *IGH* sequences that have smaller, more hydrophilic, CDR3 regions is a feature of these common vaccinations and that the clonal expansion is reduced in the older group. Given the apparent importance of the CDR3 region in this respect, it is of note that the baseline repertoire of older people comprises larger CDR3 regions, on average, than the younger group and therefore may be at a disadvantage in the initiation of appropriate immune responses.

## Experimental procedures

### Volunteers and sample collection

Young (age 18–49, *n* = 39) and older (age 65-89, *n* = 27) volunteers were recruited during the 2009/10 and 2010/11 influenza vaccination seasons from Lambeth Walk GP Practice and King's College London with informed consent and approval from the Guy's Hospital Research ethics committee. Blood and serum samples were collected prior to vaccination with the influenza (Influvac; Solvay, Southampton, UK) and 23-valent pneumococcal (Pneumovax II; Sanofi Pasteur MSD, Maidenhead, UK) vaccines and at 7 and 28 days postvaccination. PBMCs were isolated using Ficoll-Paque Plus (GE Healthcare, Buckinghamshire, UK) and Leucosep tubes (Greiner Bio-One Ltd.,Gloucestershire, UK) counted and stored in freezing medium till required. Preparation and freezing in this manner have been shown not to affect the survival of day 7 postvaccine plasmablasts (Kyu *et al.*, 2009). Samples from a subset of six young (aged 19–45, average 27 years) and six old (aged 70–89, average 78 years) subjects were chosen for *IGH* CDR3 sequencing.

### Nucleic acid extraction and cDNA conversion

Frozen PBMCs were thawed before nucleic acid extraction using the AllPrep DNA/RNA extraction kit (Qiagen, Crawley, UK). cDNA was generated from RNA using the reverse transcription system (Promega, Southampton, UK) according to manufacturer's protocol.

### Spectratypes

The CDR3 region of the rearranged *IGH* gene was amplified from cDNA using a PCR with Fw3Fam (ACACGGCTGTGTATTACTGT with a 5′-coupled Fam) and isotype-specific Cα, Cγ or Cμ primers (Cα = GGAAGAAGCCCTGGACCAGGC; Cγ = CACCGTCACCGGTTCGG and Cμ = CAGGAGACGAGGGGGAA). A 25-μL reaction mix contained 0.2 U Phusion DNA polymerase (NEB, Hitchin, UK), 200 μm of each dNTP and 0.4 μm of each primer (Fw3Fam plus one of the three isotype-specific primers) in 1× reaction buffer containing 1.5 mm MgCl_2_. PCR was amplified in 25 cycles of 98°C (30 s), 55°C (30 s) and 72°C (30 s), and 1 cycle of 72°C (5 min). Annealing temperature for Cα was 61°C.

Polymerase chain reaction samples (3 uL) were added to 1.5 uL Tamra 350 standard in formamide and run on the ABI 3730xl capillary sequencer. Data were analysed using Genescan (Applied Biosystems, Carlsbad, CA, USA) for fragment size analysis exported to Excel. These values were copied into Microsoft Excel files which were imported and analysed further in the R statistical programming environment (R Development Core Team, 2011). Raw peak data were normalised, so that the sum of all the peak heights for a sample was set to 100. A sample (*n* = 3000) was then generated from this relative frequency distribution and the mean, SD, skew and kurtosis calculated. To generate a reference Gaussian distribution, day 0 and 28 samples were combined (except for three instances where one sample showed a very low raw signal), and the mean and SD of this joint sample were used to define a normal distribution for each patient/isotype combination. The Pearson's correlation coefficient for each sample compared with the respective Gaussian distribution was then recorded as correlation to Gaussian distribution (CGD).

### Detection of antipneumococcal polysaccharide antibodies

Briefly, 96-well plates were coated overnight at 4°C with 100 μL per well of a combination of seven polysaccharide serotypes (4, 6B, 9, 14, 18C, 19F and 23F, 1 μg mL^−1^ each; ATCC, Rockville, MD, USA) in PBS with 0.02% NaN_3_. After coating, plates were washed three times with wash buffer (TBS containing 0.1% Brij 35; Sigma-Aldrich, St. Louis, MO, USA) using an automated plate washer. All serum samples were pre-absorbed with 10 μg mL^−1^ of cell wall polysaccharide (CPS; Statens Serum Institut, Copenhagen, Denmark) and 10 μg mL^−1^ of 22F polysaccharide in antibody buffer (PBS with 0.05% Tween-20) for 30 min at room temperature (RT). The reference serum standard, 89-SF, from the Food and Drug Administration (Bethesda, MD, USA), was absorbed with 10 μg mL^−1^ CPS only. The pre-absorbed serum samples and 89-SF were serially diluted and added to the microtiter plates. Plates were incubated overnight at 4°C then washed as described earlier. Alkaline phosphatase-conjugated goat anti-human IgG (Invitrogen, Paisley, UK) at a 1/10000 dilution in antibody buffer was added to the plates and incubated for 2 h at RT, and the plates were then loaded with *p*-nitrophenyl phosphate substrate (Sigma-Aldrich) in diethanolamine buffer and incubated for 2 h at RT. Reactions were stopped by the addition of 3 m NaOH, and the optical density at 405 nm was measured with a 630-nm reference using an ELISA microplate reader. The amount of antibody was determined by comparison with the 89-SF standard.

IgA and IgM plates were with HRP-conjugated goat anti-human IgA (Invitrogen) or IgM antibodies (Thermo Fisher, Huntsville, AL, USA) at 1/4000 in antibody buffer for 2 h at RT. Plates were detected with TMB chromogen substrate (Invitrogen), and OD was read at 450 nm.

### Statistics

Statistics were performed with GraphPad Prism 4.0 (Graphpad Inc, La Jolla, CA, USA) using Mann–Whitney *u*-test to compare groups, chi-squared to compare frequencies and Pearson's correlation analysis to test for association between different metrics.

### IgG ANA

Nonorgan-specific autoantibodies (ANA) were screened by indirect immunofluorescence on HEp-2 cells. By using the fully automated reading system AKLIDES (Hiemann *et al.*, 2009) for screening and preclassification of ANA, we could minimise variation of detected intensities.

### Sequencing

High-throughput sequencing of *IGHV* genes was performed as previously described (Wu *et al.*, 2010). Briefly, *IGHV* genes were amplified using a seminested, isotype-specific PCR, with primers containing multiplex identifier (MID) sequences in the second round of amplification. To produce sufficient DNA for sequencing, while minimising PCR amplification, PCR repeats were performed for each individual experimental sample. PCR primers were removed from the pooled products by electrophoresis and using QIAquick gel purification kit. Samples to be pooled for sequencing were mixed in equal quantities and concentrated using QIAquick PCR purification kit (Qiagen) before sequencing on the GS FLX Titanium sequencer (Agowa GmBH, Berlin, Germany). Accuracy of the method as a whole was previously determined to be less than one error per 1300 bp (not counting indels). After sequencing, the sequences were subjected to stringent quality controls and assigned to the corresponding samples based on the terminal MID sequence. The amino acid sequence of each CDR3 junction region was determined using V-Quest, allowing for indels. The physicochemical properties of the peptide between the conserved first and last amino acid positions were determined using ProtParam. Clonally related sequences were identified based on the DNA sequence of the CDR3 (‘junction’) region as reported by V-Quest. A distance matrix between all pairwise comparisons was created as follows. For each sequence pair, the shorter sequence was defined as the test sequence. If the other sequence was 10 or more nt longer, or belonged to a different *IGHV* gene family, the distance was set at 1 (unrelated). Otherwise, the distance between the sequences was defined as minimum edit distance normalised to the length of the test sequence, upto a maximum of 1 in 5 (or a total of 10) mismatches or indels. This distance matrix was then used for hierarchical clustering (complete method), with clones being defined by cutting the resultant dendrogram at a distance of 0.25.

## Supporting Information

**Fig. S1** Virtual spectratypes without the effect of clonal expansion.

**Fig. S2** Large clonal expansions at small CDR3 sizes in the young group only.

**Fig. S3** Smaller CDR3 regions are more hydrophilic.

**Fig. S4** Relationship between spectratype metrics at day 7 after vaccination and PPS-specific antibody.

**Fig. S5** CDR3 size before vaccination does not correlate to specific pneumococcal vaccine levels.

As a service to our authors and readers, this journal provides supporting information supplied by the authors. Such materials are peer-reviewed and may be re-organized for online delivery, but are not copy-edited or typeset. Technical support issues arising from supporting information (other than missing files) should be addressed to the authors.
