# Supplementary figures and images for: Vaccination-induced changes in human B-cell repertoire and pneumococcal IgM and IgA antibody at different ages
Source: Aging Cell. 2011 Dec;10(6):922–30. doi: 10.1111/j.1474-9726.2011.00732.x (PMC3264704; doi:10.1111/j.1474-9726.2011.00732.x)

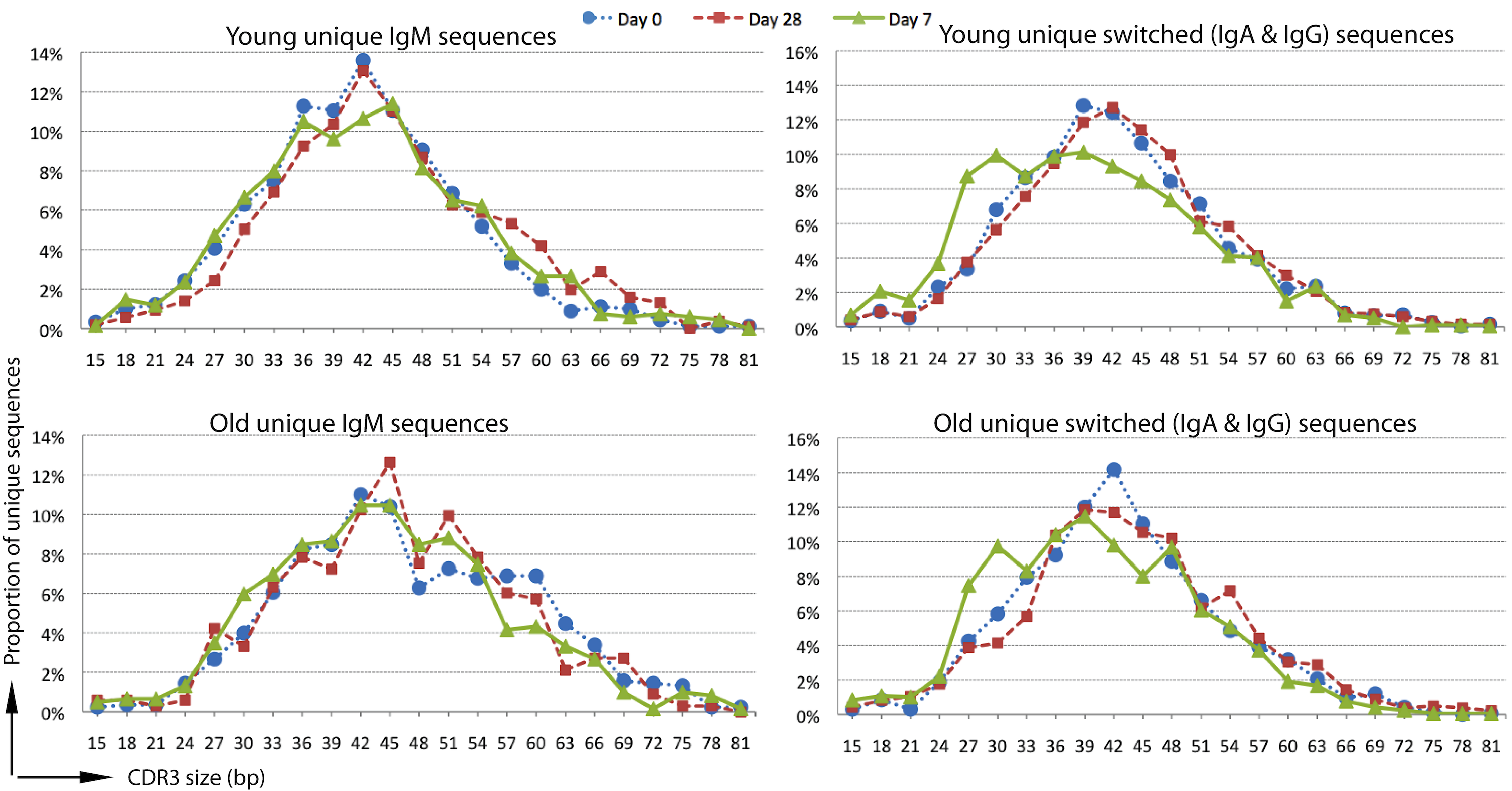

Supplement: Supplementary file 1 [file acel0010-0922-SD1.tiff]

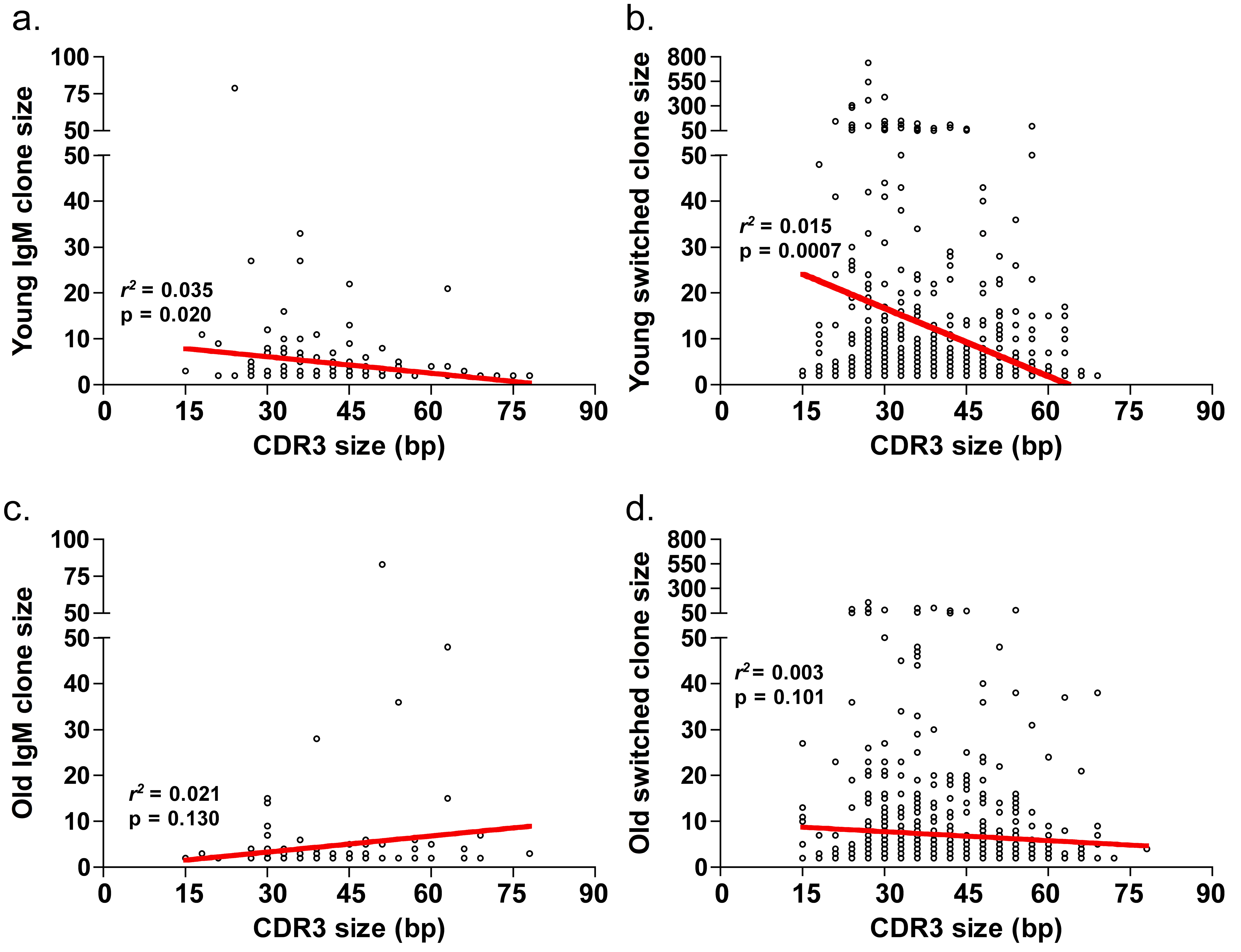

Supplement: Supplementary file 2 [file acel0010-0922-SD2.tif]

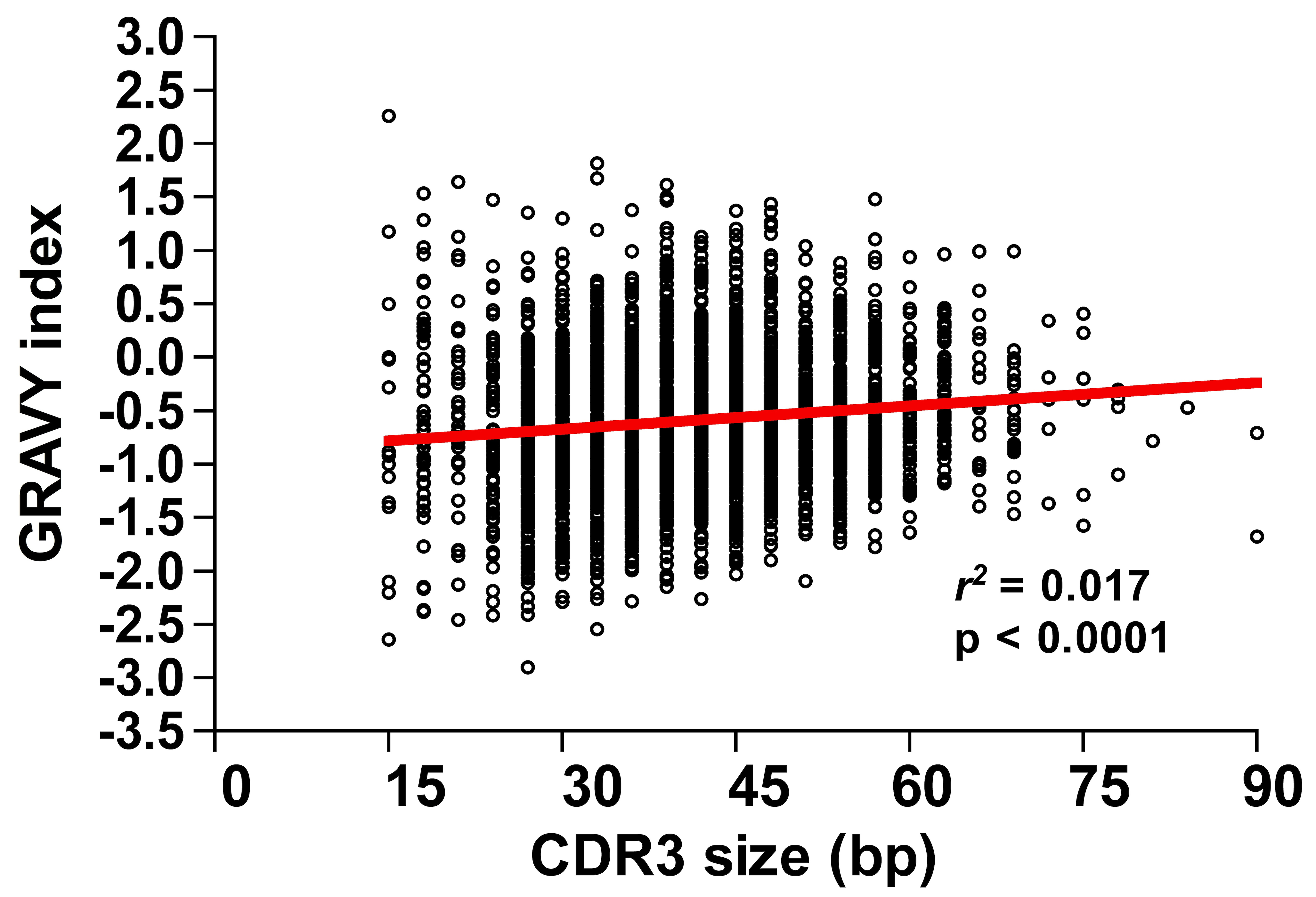

Supplement: Supplementary file 3 [file acel0010-0922-SD3.tiff]

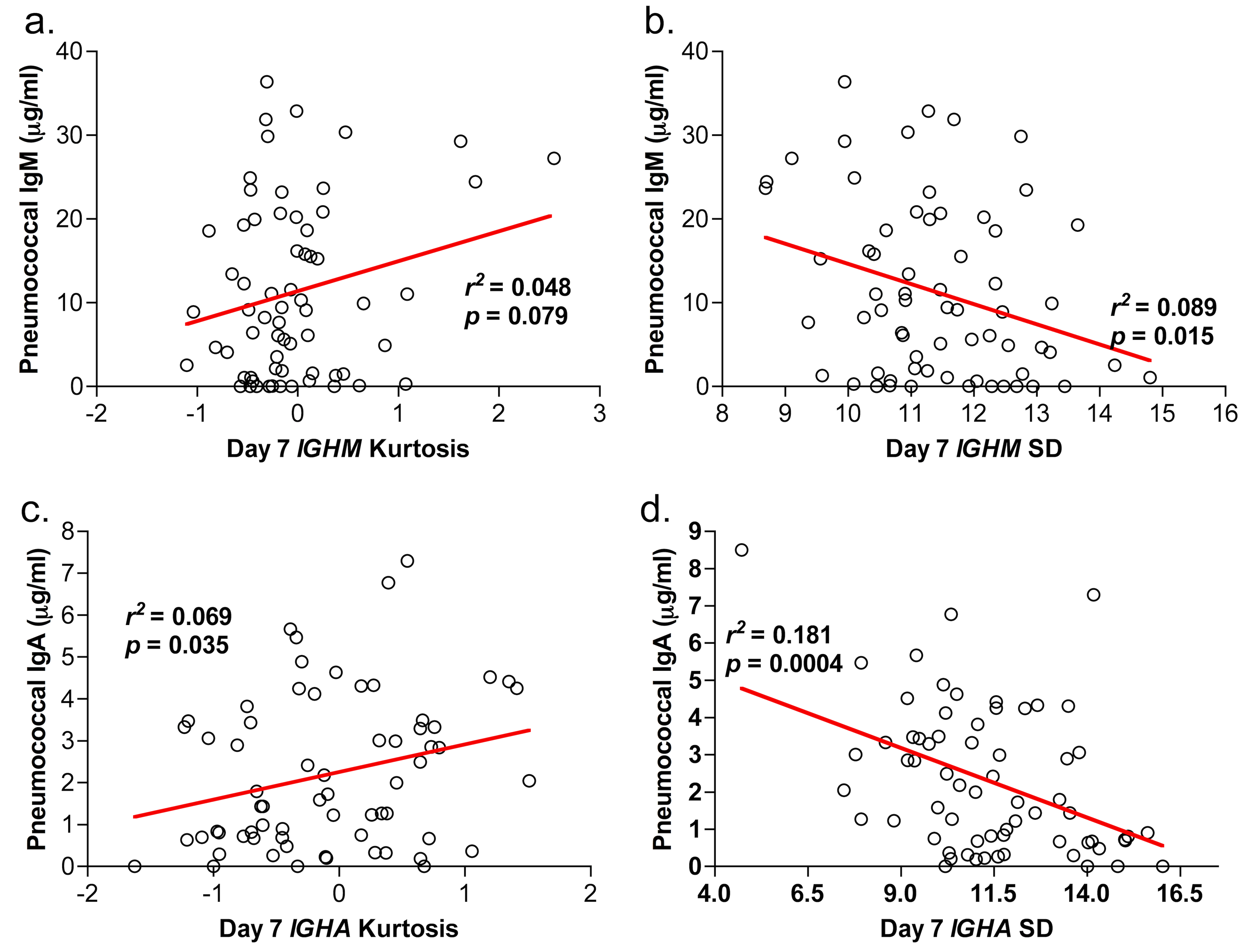

Supplement: Supplementary file 4 [file acel0010-0922-SD4.tif]

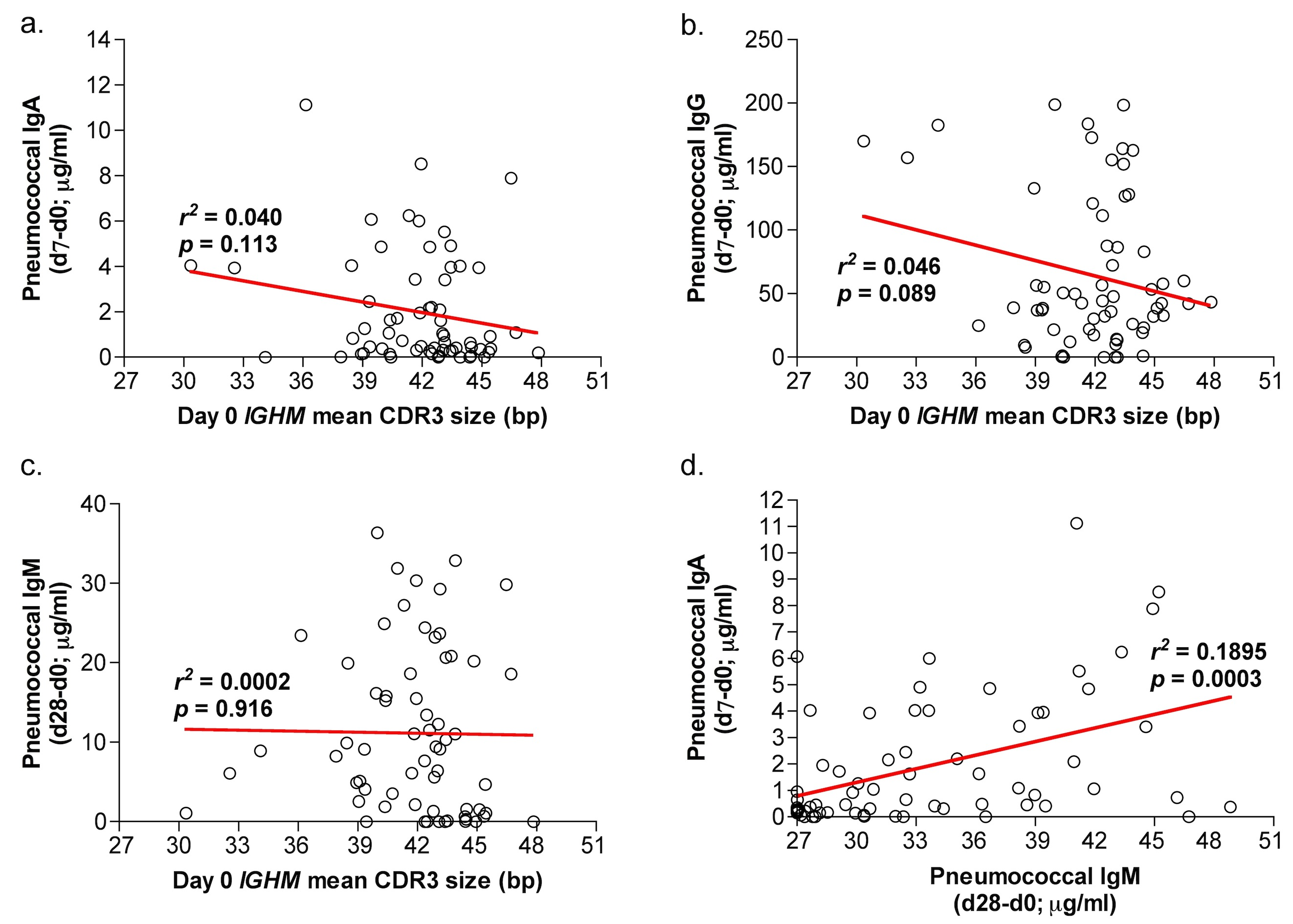

Supplement: Supplementary file 5 [file acel0010-0922-SD5.tiff]
